# Pleiotropic Effects of Cardiac Resynchronization Therapy on Cardiometabolic Modulation in Heart Failure

**DOI:** 10.3390/medicina62030465

**Published:** 2026-02-28

**Authors:** Panagiotis Theofilis, Panagiotis Iliakis, Aikaterini-Eleftheria Karanikola, Michail Botis, Kyriaki Mavromoustakou, Panagiotis Xydis, Nikolaos Ktenopoulos, Paschalis Karakasis, Ioannis Leontsinis, Christina Chrysohoou, Konstantinos Tsioufis

**Affiliations:** 1 First Department of Cardiology, National and Kapodistrian University of Athens, Hippokration General Hospital of Athens, 115 27 Athens, Greecepanayiotisiliakis@gmail.com (P.I.); elinakaranikola@gmail.com (A.-E.K.); nikosktenop@gmail.com (N.K.);; 2 Second Department of Cardiology, Aristotle University of Thessaloniki, Hippokration General Hospital of Thessaloniki, 541 24 Thessaloniki, Greece

**Keywords:** cardiac resynchronization therapy, HF, gut microbiome, cardiometabolic

## Abstract

Cardiac resynchronization therapy (CRT) is a cornerstone intervention for patients with heart failure (HF) and electrical dyssynchrony, improving quality of life, functional capacity, and survival. Beyond mechanical synchrony, mounting evidence suggests CRT exerts systemic and myocardial cardiometabolic benefits. CRT acutely enhances mechanical efficiency and shifts substrate utilization toward greater oxidation of fatty acids and ketones, effects that correlate with long-term reverse remodeling on cardiac magnetic resonance imaging. Earlier metabolomic profiling demonstrated that CRT normalizes circulating energy metabolites, improving Krebs cycle intermediates and substrate balance between glucose and lipids, while baseline metabolite patterns may differentiate responders from non-responders. These metabolic adaptations accompany favorable changes in diastolic performance, right ventricular function, and ventriculo-arterial coupling. In parallel, improved splanchnic perfusion and reduced congestion may ameliorate gut dysbiosis and endotoxemia, mitigating systemic inflammation. Collectively, these findings position CRT as a therapy capable of both mechanical and metabolic restoration in advanced HF. In this review, we discuss the emerging data on how CRT reconditions myocardial energy metabolism, influences ventricular–arterial interactions, and modulates peripheral and gut-derived metabolic pathways.

## 1. Introduction

Heart failure (HF) is a major global health burden, characterized by high morbidity, mortality, and health-care costs. Although initially attributed to impaired pump function and elevated filling pressures, HF’s pathophysiology extends well beyond, as it is accompanied by a maladaptive cardiometabolic milieu [1], consisting of sympathetic and renin–angiotensin–aldosterone system (RAAS) overactivation, endothelial dysfunction, inflammation, adverse adipokine signaling, mitochondrial/oxidative stress, and shifts in myocardial substrate utilization [2,3,4,5,6]. The dysregulation of those pathways may provide the ground for worsening congestion, impaired quality of life (QoL), and, ultimately, adverse clinical outcomes.

Over the past decades, the evolution of pharmacotherapy has led to significant improvement in the QoL and prognosis of affected patients [7]. In addition, device therapies have gained ground in the therapeutic arsenal of HF, namely cardiac resynchronization therapy (CRT), which is a foundational device therapy for patients with symptomatic HF, reduced left ventricular ejection fraction (LVEF), and electrical dyssynchrony. By coordinating activation of the right and left ventricles via biventricular pacing or, when appropriate, physiologic conduction system pacing [8], CRT restores intra- and interventricular timing, increases forward stroke volume, lowers wall stress, and reduces functional mitral regurgitation, culminating in reverse remodeling and fewer HF hospitalizations and deaths in appropriately selected patients [9,10]. Benefit is greatest with classic left bundle branch block and wide QRS (≥150 ms), whereas responses are attenuated in non-LBBB morphologies or intermediate QRS durations (120–150 ms), underscoring the need for precise electrophysiologic phenotyping. Indications have expanded from de novo implants to upgrade scenarios (e.g., pacing-induced cardiomyopathy from high RV pacing burden), and device choice (CRT–pacemaker (CRT-P) vs. CRT–defibrillator (CRT-D)) is individualized by arrhythmic risk, myocardial scar burden, and competing comorbidities. Yet heterogeneity of response persists, often ~30% by conventional reverse remodeling criteria [11].

CRT’s hemodynamic gains plausibly propagate beyond mechanical synchrony to recondition systemic biology. Improved forward flow and relief of venous/organ congestion can attenuate sympathetic and RAAS overdrive, enhance renal perfusion and natriuresis, and lessen hepatic–splanchnic congestion, upstream shifts that intersect with metabolic pathways central to HF. Specifically, CRT may recalibrate energy handling at myocardial and peripheral levels, modify diastolic function, right ventricular (RV) performance, and ventriculo-arterial coupling through load redistribution and autonomic/vascular effects. Moreover, it can potentially influence the gut–heart axis by improving splanchnic perfusion and intestinal barrier integrity, mechanisms with potential downstream impact on microbial translocation, inflammatory tone, and metabolite profiles.

In this review, we aim to highlight the beneficial cardiometabolic effects of CRT in the setting of HF, ranging from myocardial energy handling, amelioration of diastolic function and ventricular–arterial coupling, and its impact on the gut microbiome. Moreover, we discuss the alterations in the cardiometabolic profile following CRT implementation.

## 2. Clinical Benefits of CRT

Over the past two decades, a series of randomized controlled trials (RCTs) and observational studies have demonstrated that CRT improves both quality of life and morbidity when the population is selected properly (Table 1). Pioneer trials such as the smaller MUSTIC and, more importantly, the MIRACLE established proof of concept that biventricular pacing improves symptoms, exercise capacity, and QoL in patients with advanced HF. The MIRACLE trial (NYHA III–IV, reduced LVEF, wide QRS) demonstrated significant improvements in the Minnesota HF Questionnaire scores, 6 min walk distance, and NYHA functional class compared with optimal medical therapy alone (OMT). These results confirmed that CRT not only improved echocardiographic parameters but could change lives [12,13]. In the COMPANION trial, patients with advanced HF were randomized to medical therapy, CRT with pacing only (CRT-P), or CRT with defibrillation (CRT-D). Both CRT arms significantly reduced the composite of death or first hospitalization compared with OMT, while CRT-D also achieved a significant reduction in all-cause mortality [14]. The CARE-HF trial [15] came as the cornerstone trial by providing evidence for CRT’s impact on morbidity and mortality. Among patients with NYHA III–IV symptoms, cardiac dyssynchrony, and reduced LVEF despite OMT, CRT reduced the interventricular mechanical delay, the end-systolic volume index, and the area of the mitral regurgitant jet. In parallel, it increased left ventricular ejection fraction and improved symptoms and the quality of life. CARE-HF clearly suggested implantation of a CRT device should routinely be considered in the above population.

After the above optimistic results, subsequent trials evaluated CRT in milder HF stages. MADIT-CRT enrolled predominantly NYHA I–II patients with reduced LVEF who were ICD candidates [16]. CRT-D reduced HF events by approximately 30–40% compared with ICD alone, driven by fewer hospitalizations. Mortality rate differences were not statistically significant. The benefits were most pronounced in patients with LBBB and QRS ≥150 ms, which remains the favorable combination.

The RAFT trial (2010) extended these findings, showing that CRT-D reduced the risk of death or HF hospitalization and produced long-term mortality benefits [17]. For the first time, though, higher device-related complication rates were discussed.

The REVERSE trial focused on structural and functional remodeling in mild HF [18]. CRT led to significant left ventricular reverse remodeling and delayed HF progression, but there was no difference in mortality. The reverse LV remodeling remained stable even after 5 years. The role of this therapy among patients with mildly symptomatic HF remains less impressive. These trials demonstrated that earlier CRT implantation may slow disease progression, even when symptom gains are modest.

Modern medication additions like mineralocorticoid receptor antagonists, sacubitril valsartan, and sodium-glucose co-transporter 2 inhibitors have significantly improved mortality rates, so the impressive benefit of CRT probably requires longer follow-up [19]. The combined effect of electrical and medical therapy with mechanisms may explain the impressively low mortality rate in the biggest CRT trial to date (AdaptResponse trial), which included 3617 patients in NYHA classes II–IV.

Meta-analyses during the last decade consistently continue to confirm that CRT improves QoL, exercise capacity, and functional status while reducing HF hospitalizations and all-cause mortality in appropriately selected patients [20]. In contrast, patients without favorable features, with significant RV dysfunction, or with narrow QRS derive less benefit. In 2024, Fudim M et al. highlighted that CRT significantly reduces HF hospitalizations and mortality across comorbidity groups, emphasizing its durable benefit even in complex patients [21]. Friedman and colleagues pooled trial data demonstrating that CRT improves outcomes primarily in patients with LBBB or broad QRS complexes, underscoring that the importance of the QRS duration criterion remains [22].

Further publications evaluated CRT in specific subgroups. Meta-analyses in atrial fibrillation (AF) populations found attenuated benefit unless biventricular pacing was ensured [23]. Focusing on AF patients, CRT did not decrease mortality compared with ICD or medical therapy alone in patients with HF and AF with indications for CRT [24]. Interestingly, there was no difference in all-cause mortality in CRT-AF and AVJ ablation patients compared with CRT patients with normal sinus rhythm.

Despite the above-mentioned benefits of CRT, it should be noted that approximately one-quarter of CRT recipients are “non-responders,” underscoring the importance of careful patient selection and optimal lead positioning, although in many cases the reason is far more complex. The “non-response” phenomenon should not be considered as a dichotomous event but rather as a spectrum of outcomes that may be influenced by various factors [25]. AF, advanced stage of HF, ischemic etiology—especially with extensive fibrosis—non-LBBB ECG pattern, as well as apical and anterior position of LV lead, may contribute to reduced resynchronization [25,26]. Moreover, due to a slightly more complex procedure sequence and duration, an increased rate of complications has been seen, namely LV lead dislodgement, cardiac tamponade, infection, and loss of biventricular pacing [27]. In light of the above, it is crucial to identify the population most likely to respond in order to avoid unnecessary risks. CRT is a class I recommendation for LVEF ≤ 35%, LBBB with QRS duration ≥ 150 ms, sinus rhythm, and NYHA class II symptoms and above [28]. As far as the rest of the indications are concerned, the possibility of non-response should be taken into account. On the other hand, an improved response can be achieved through programming adjustments [29,30]. Therefore, an initial moderate effect should not be discouraging.

## 3. Effects of CRT on Energy Handling

From a molecular standpoint, dyssynchronous contraction in the failing heart (DHF) is characterized by downregulation of Ca^2+^ handling proteins and connexin 43, as well as upregulation of mitogen-activated protein kinase (MAPK), in the lateral wall only [31]. Similar experimental models demonstrate an increase in p38 MAPK and Ca^2+^-calmodulin kinase II (CaMKII) activation in the lateral wall of DHF ventricles. The latter effect was reversed by CRT utilization [32]. Importantly, p38 MAPK is a stress-activated signaling pathway involved in inflammatory responses, fibrosis, and apoptosis, while CaMKII regulates Ca^2+^ handling and excitation-contraction coupling and is associated with apoptosis and cardiac hypertrophy through β-adrenergic signaling. In dyssynchronous HF, regional overactivation of these kinases contributes to adverse remodeling [33,34].

The deleterious effects of asynchronous left ventricle activation extend to the electrophysiological aspect. Action potential (AP) prolongation is most prominent in cells originating from the late-activated lateral left ventricle wall and is a marker of heterogeneous electrical properties in the failing heart. CRT shortens the AP in lateral myocytes and subsequently reduces LV regional heterogeneity in canine models [35].

The stress response kinases and electrophysiological dysregulation seem to represent a broader transcriptomic dysregulation in the DHF. In global gene expression profiling studies performed in canine models, six times as many genes were identified to be differentially expressed between nonfailing and DHFs in anterior compared with lateral LV myocardium (2173 versus 346 transcripts, respectively; false discovery rate < 5%) [36]. Specifically, prominent downregulation of metabolic pathways, including oxidative phosphorylation, fatty acid, amino acid, and glycose metabolism, was noted. Landmark human trials come in agreement with these conclusions. Nowak et al. demonstrated, through gated PET with 18F-fluorodeoxyglucose and 99mTc-sestamibi single-photon emission-computed tomography, that glucose metabolism is reduced more than perfusion in the septal compared with the LV lateral wall in patients with dilated cardiomyopathy and LBBB [37]. The proposed pathophysiologic mechanism is that in LBBB, early septal activation produces contraction against an unopposed, relaxed LV free wall, yielding limited wall stress and negligible ejection, as intraventricular pressure is still low [38]. In contrast, the later-activated LV lateral wall contracts under higher mechanical load, as chamber pressure has already risen.

Mitochondrial function seems to be favorably altered by CRT. Agnetti et al. [39] demonstrated that mitochondrial respiration and efficiency of oxidative phosphorylation are restored and improved by CRT. The favorable effects were not limited in the respiratory chain (all complexes of oxidative phosphorylation were favored, apart from complex IV) but exceeded to the metabolic pathways supplying the substrates and key enzymes involved in the Krebs cycle, namely pyruvate carboxylase, pyruvate dehydrogenase, and E1 and E2 subunits, as well as aldehyde dehydrogenase, α-keto acid dehydrogenase E2, and ferredoxin reductase. In parallel, CRT reversed the depression of mitochondrial oxidative efficiency (ADP/O_2_), presumably by augmenting mitochondrial reactive oxygen species–scavenging proteins. Notably, these mitochondrial effects have not been reproduced by pharmacological HF therapies.

**Figure 1:**
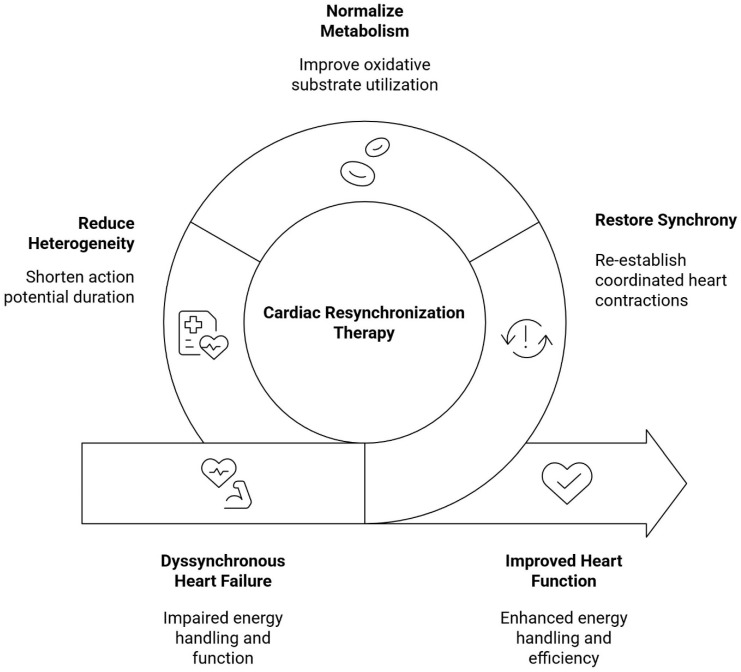
Overview of the cardiometabolic and functional effects of CRT.

The latest human study using paired arteriovenous sampling and pressure–volume analysis shows that CRT acutely increases stroke work without raising myocardial O_2_ consumption and promptly shifts substrate handling toward greater uptake of long- and medium-chain fatty acids and ketones under both insulin/glucose and intralipid clamps [40]. Importantly, the magnitude of these acute substrate-uptake responses correlates with 6-month reverse remodeling on cardiac MRI, and this relationship appears independent of the degree of acute QRS shortening, emphasizing that metabolic flexibility, rather than electrical narrowing per se, tracks longer-term structural recovery. These data argue that CRT not only normalizes mechanics but also reprograms myocardial energetics in a direction associated with chamber reverse remodeling. Prior metabolomic profiling complements this mechanistic link: in a prospective cohort, CRT moved the circulating metabolome toward a more physiologic state, with improvements in Krebs cycle indices and a shift in the glucose–to–palmitate balance, while baseline metabolite signatures differentiated responders from non-responders [41]. Together, these findings support a model in which CRT responsiveness depends on a myocardium capable of uptitrating oxidative substrate use once mechanical inefficiency is relieved (Figure 1).

## 4. Effects of CRT on Diastolic Function, RV, and Aorto-Ventricular Handling

Conflicting data exists on the effect of CRT on LV diastolic function. CRT seems to improve several load-dependent parameters, mainly by reducing mitral E velocity, E/A ratio, E/Vp ratio, and filling pressures (E/Em), while increasing deceleration time and diastolic filling time, but it shows no significant effect on load-independent tissue Doppler indices of relaxation [42]. Earlier reports suggest that LV diastolic filling parameters improve in CRT responders within four months of the procedure in conjunction with LV systolic function improvement, whereas LV relaxation parameters remain unaffected [43]. On a similar note, Facchini et al. reported that despite apparent LV reverse remodeling and improved filling characteristics at 4 months, CRT did not significantly affect relaxation and filling pressures in patients with systolic HF [44]. On the other hand, Jansen et al. observed that both load-dependent and relatively load-independent indices of diastolic function improved significantly after CRT, but only in patients exhibiting LV reverse remodeling, with grade 2 and 3 dysfunction decreasing from 34% to 13% [45]. Shanks et al. further demonstrated improved diastolic synchrony among CRT responders acutely and at 6 months after the procedure, reflected by the maximal time delay in peak early diastolic velocities in basal LV segments on tissue Doppler imaging [46]. Novel evidence suggests that CRT also significantly improves LA reservoir strain (LASr), a parameter closely related to ventricular filling pressures and diastolic function [47,48].

Initial findings from pivotal CRT clinical trials yielded contradictory evidence regarding the impact of resynchronization therapy on RV function. In the MADIT-CRT trial, among 1273 patients with mild HF symptoms, RV function, as assessed by fractional area change (FAC), improved by 8.1 ± 5.5% in the CRT-D group compared with 5.4 ± 4.8% in the ICD group (*p* < 0.001), in parallel with improvements in LV ejection fraction (LVEF) and contributing to a reduced incidence of all-cause mortality and HF events [49]. In contrast, subanalyses from the CARE-HF and REVERSE trials found no significant changes in tricuspid annular plane systolic excursion (TAPSE) with CRT [50,51]. However, several pooled analyses suggest an overall favorable effect of CRT on RV function. In a meta-analysis of 13 studies including 1541 patients, Sharma et al. [52] reported significant improvements in both anatomical and functional RV parameters, including RV dimensions, TAPSE, S’, and FAC, findings further supported by a more recent meta-analysis of 30 studies demonstrating similar improvements after 6 to 12 months of follow-up [53].

In recent years, numerous studies have focused on the effects of CRT on ventriculo-arterial coupling (VAC). VAC represents a key combined marker of cardiac and vascular function, whose assessment—traditionally via the Ea/Ees ratio for the LV—offers invaluable diagnostic, prognostic, and therapeutic insights across the whole HF spectrum and multiple common comorbidities [54]. Studies focusing both on invasive and non-invasive/echocardiographic measurements have demonstrated a favorable effect of resynchronization through biventricular pacing on LVAC, both in the acute and chronic post-procedural phase [55,56,57]. Notably, multipoint LV pacing has also exhibited remarkable effects compared to traditional, optimized CRT in the improvement of LV hemodynamic parameters [29]. More recently, RV-to-pulmonary artery coupling (RVAC) has emerged as a valuable predictor of HF prognosis and can be noninvasively assessed using the TAPSE/Pulmonary Artery Systolic Pressure (PASP) ratio in transthoracic echocardiography [58]. CRT has been shown to improve R within the first 6 months after the procedure in a cohort of 31 patients [59]. However, other evidence concluded that RVAC improvement occurs mainly in the subgroup of CRT responders [60,61].

## 5. Effects of CRT on Gut Microbiome

The gut microbiota refers to the collection of microorganisms (bacteria, viruses, fungi, and archaea) that live in the digestive tract, primarily in the intestines, while the gut microbiome describes the entire genetic makeup of the gut microbiota, encompassing all microbial genes and their functions. Evidence indicates that the gut microbiome plays a crucial role in the pathophysiology of HF. Although the bidirectional relationship between HF and the gut microbiota is not yet fully understood, studies suggest that bacterial translocation in HF patients results from multiple mechanisms that lead to both functional and structural changes in the gastrointestinal tract as part of systemic compensatory responses and immune dysregulation [62].

Intestinal ischemia due to reduced perfusion in HF leads to alterations in pH, tissue hypoxia, epithelial dysfunction, edema, and increased intestinal wall permeability [63,64]. This increased permeability facilitates the translocation of bacteria and bacterial toxins, such as lipopolysaccharides (LPS), from the gut into the systemic circulation [65]. LPS, a biologically active component of Gram-negative bacteria, can activate the toll-like receptor 4 (TLR4), triggering immune responses. Studies have shown elevated LPS levels in the hepatic veins of HF patients, supporting the hypothesis of microbial translocation [66]. The role of LPS appears to be bidirectional, not only as a consequence of HF but also as a contributor to the progressive deterioration of cardiac function via further disruption of the intestinal barrier [67].

The absorption of endotoxins into the systemic circulation stimulates the production of pro-inflammatory cytokines [68]. Recent studies have demonstrated a strong correlation between HF and a chronic inflammatory state, which can be triggered or exacerbated by bacterial translocation, contributing to myocardial dysfunction [69]. Elevated cytokine levels are associated with more severe clinical symptoms and worse prognosis in HF patients [70]. In cases of compensated HF, LPS levels are linked to increased inflammatory markers, which tend to decrease following clinical decompensation [71].

Metabolites produced in the intestine may serve as potential biomarkers for HF, reflecting the role of intestinal dysbiosis in disease progression [28,72]. Metabolites such as trimethylamine N-oxide (TMAO), short-chain fatty acids (SCFAs), phenylalanine, and ricinoleic acid have been associated with inflammation, cardiac dysfunction, and prognosis in HF patients [28,72,73]. TMAO is linked to myocardial fibrosis, while SCFAs and glycine appear to exert protective effects on the myocardium. In patients with HF, increased levels of pro-inflammatory and decreased levels of anti-inflammatory metabolites have been observed. These findings support the hypothesis that the gut microbiome and its metabolites could be used as prognostic indicators in HF patients [28,72,73,74,75,76].

At present, no studies have demonstrated a direct relationship between cardiac resynchronization therapy and gut microbiome remodeling. However, improvements in cardiac function following CRT implantation, as described above, may secondarily contribute to enhanced gut barrier integrity and more favorable profiles of microbe-derived metabolites. Therefore, any potential changes in the gut microbiome observed after CRT should be interpreted as indirect consequences of improved cardiac function and hemodynamic status, rather than as a direct effect of the therapy itself.

## 6. Effects of CRT on Cardiometabolic Profile

CRT induces significant neurohormonal and autonomic changes beyond mechanical resynchronization that contribute to systemic cardiometabolic regulation in patients with HF [14]. Improvements in autonomic balance following CRT are likely mediated, at least in part, by reflex mechanisms secondary to enhanced hemodynamic performance. While these changes may not represent strictly pleiotropic effects, reduced sympathetic activation may nonetheless contribute to systemic cardiometabolic regulation in patients with HF. Sympathetic nervous system overdrive is a key deleterious component of the failing heart’s pathophysiology [77]. This has been highlighted by evidence showing that treatment with sodium-glucose co-transporter 2 inhibitors is associated with amelioration of sympathetic tone [78]. By restoring ventricular dyssynchrony and improving cardiac output, CRT reduces chronic sympathetic activation, a key driver of insulin resistance, lipolytic dysregulation, and adverse metabolic remodeling in HF. Clinical studies have demonstrated significant reductions in circulating norepinephrine levels and improved autonomic balance following CRT implantation, particularly among patients classified as CRT responders [79]. In parallel, CRT ameliorates RAAS’s overdrive, resulting in reduced aldosterone and renin activity, which are implicated in visceral adiposity, insulin resistance, and impaired glucose utilization [80]. Improvements in heart rate variability after CRT further support enhanced parasympathetic modulation, with these autonomic changes correlating with improved exercise capacity and metabolic flexibility [81]. Preclinical models of dyssynchronous HF demonstrated that restoration of electrical synchrony normalizes myocardial β-adrenergic signaling activity, reduces oxidative stress, and improves mitochondrial efficiency, mechanisms that indirectly influence systemic metabolic homeostasis [82,83,84]. Importantly, the magnitude of neurohormonal suppression appears proportional to the hemodynamic response to CRT, underscoring the central role of CRT response in mediating downstream cardiometabolic effects [26,85].

HF is frequently accompanied by insulin resistance and impaired glucose metabolism, driven by reduced skeletal muscle perfusion, chronic sympathetic activation, inflammation, and mitochondrial dysfunction [86]. Clinical observational studies suggest that CRT is associated with improvements in insulin sensitivity, particularly among CRT responders and patients with type 2 diabetes mellitus [41,87]. In a small prospective cohort, reductions in fasting plasma glucose and glycated hemoglobin (HbA1c) levels have been reported within months following CRT implantation, paralleling improvements in functional capacity and reverse ventricular remodeling [88]. However, available evidence, mainly limited by small sample sizes, heterogeneity in metabolic endpoints, and short follow-up, remains preliminary and appears mainly confined to CRT responders and specific subgroups, highlighting the need for larger prospective studies [17].

Regarding lipid-related metabolic parameters, available studies have primarily focused on circulating lipid profiles, including total cholesterol, low-density lipoprotein cholesterol (LDL-C), high-density lipoprotein cholesterol (HDL-C), and triglyceride levels. HF is commonly accompanied by impaired fatty acid oxidation and altered lipoprotein profiles, and CRT-mediated hemodynamic improvement may secondarily modulate these pathways through enhanced organ perfusion and reduced neurohormonal stress [41,89]. Preclinical data even demonstrated that the alteration of lipid metabolism precedes the development of HF in hypertensive rats [90]. At the myocardial metabolic level, Green et al. demonstrated that CRT implantation was associated with a 48% (*p* < 0.001) decrease in LV end-diastolic volume, being correlated with increased fatty acid uptake and increased ketone uptake (*p* = 0.05) [40]. As highlighted by the authors, this metabolic flexibility of the myocardium is driven by the true reverse remodeling 6 months post-implantation.

In addition to lipid profiles, some studies have explored changes in broader metabolic or oxidative stress-related markers, such as serum uric acid and bilirubin levels, although their relationship to CRT remains insufficiently characterized. CRT implantation and its direct and indirect hemodynamic improvement may partly ameliorate congestion, associated with benefits in hepatic function (thereby altering lipoprotein synthesis and clearance); these are clinically relevant, as they underscore that CRT response can influence cardiometabolic biomarkers beyond classic echocardiographic endpoints [88]. All aforementioned evidence was vividly shown by Boros et al., who enrolled 129 HF patients who underwent CRT implantation and 120 controls; routine laboratory examination was carried out at baseline, 6 months, and 2 years post-implantation [88]. Glucose, serum uric acid, and total bilirubin and glucose levels were statistically significantly decreased in 6 months.

More granular evidence arises from metabolomics, where CRT has been associated with adaptive transitions in circulating metabolites spanning lipid pathways, including shifts in indices reflecting the balance between glycolytic and fatty acid metabolism after implantation [41]. Notably, metabolomic signatures differed between CRT responders and non-responders, suggesting that favorable “lipid substrate” patterns may track with reverse remodeling and could potentially contribute to risk stratification [41].

Overall, current evidence indicates limited and heterogeneous lipid-related metabolic changes following CRT, observed mainly among responder populations and likely secondary to improvements in hemodynamic performance and autonomic balance [41,88].

## 7. Beyond the Heart: Renal and Hepatic Effects of CRT

Renal dysfunction in HF is often mediated not only by reduced renal arterial perfusion but also by renal venous congestion (elevated central venous and renal interstitial pressures), which can impair glomerular filtration and contribute to diuretic resistance [91]. CRT may beneficially modulate the cardio-renal axis through combined effects on forward flow (improved cardiac output and reduced functional mitral regurgitation) and decongestion (lower filling pressures) [92], thereby improving renal perfusion gradients and natriuresis in responsive phenotypes. In a post hoc analysis of the randomized, placebo-controlled MIRACLE trial, CRT improved renal indices specifically in patients with moderately reduced baseline estimated glomerular filtration rate (eGFR), with eGFR increasing in the CRT arm while declining in controls and with a concordant improvement in BUN, whereas no significant between-group differences were observed in patients with preserved/mildly impaired baseline renal function [93].

Consistent with this pattern, observational data similarly suggest renal stabilization or improvement after CRT, particularly among responders. In a cohort study examining CRT recipients across chronic kidney disease (CKD) stages, mean GFR remained stable overall over follow-up, while patients with advanced CKD demonstrated significant improvement; importantly, stable or improved GFR independently predicted mortality after multivariable adjustment, supporting renal trajectory as a clinically meaningful marker of systemic response [94]. A smaller study also reported that preservation or improvement in eGFR is concentrated among CRT responders, paralleling functional and echocardiographic gains and facilitating optimization of guideline-directed medical therapy, including higher utilization and/or up-titration of renin–angiotensin system blockers and β-blockers in responders [95]. Overall, the literature suggests that CRT’s renal impact is heterogeneous and appears strongest when renal dysfunction is at least partly hemodynamic (congestion/low output) and when patients are CRT responders. These findings support monitoring renal trajectories after CRT as an adjunct marker of systemic benefit and as a potential enabler for optimization of HF pharmacotherapy.

As with renal dysfunction, liver dysfunction in HF results from hepatic hypoperfusion from reduced cardiac output and hepatic venous congestion from elevated right-sided filling pressures/central venous pressure, which may manifest as congestive hepatopathy with predominantly cholestatic abnormalities (bilirubin, GGT) and impaired synthetic function (albumin). Clinical evidence supports that hepatic biomarkers can improve after CRT in parallel with systemic reverse remodeling. In a longitudinal biomarker study of CRT recipients, baseline liver enzymes and total bilirubin were elevated, and albumin was reduced in HF patients, with significant reductions in AST/ALT/GGT and increased albumin observed during longer-term follow-up after CRT [88]. Complementing this, Hosoda et al. demonstrated that the direction of bilirubin changes during early follow-up after CRT carries mechanistic and prognostic information [96]. Patients with decreased bilirubin showed significant LV reverse remodeling and LVEF improvement, whereas those with increased bilirubin did not [96]. Moreover, increased bilirubin independently predicted the composite of cardiac mortality and HF hospitalization [96]. Taken together, current evidence supports that CRT-mediated hemodynamic improvement and decongestion can be accompanied by improvement in hepatic biomarkers in responsive patients.

## 8. CRT and Digital Health: Remote Monitoring to Maximize Systemic Benefit

Digital health integration is increasingly relevant to CRT pathways because the pleiotropic benefits of CRT depend on sustained delivery of effective biventricular pacing and timely recognition of decompensation. Remote device monitoring can facilitate early identification of factors that reduce biventricular pacing percentage, such as atrial tachyarrhythmias, and enable targeted interventions to preserve CRT exposure [97]. Contemporary CRT systems support wireless data transmission to manufacturer repositories via landline or cellular networks, enabling continuous surveillance of device integrity and clinically relevant events in addition to scheduled remote interrogations. From a HF management standpoint, remote monitoring aims to detect worsening congestion before overt clinical deterioration, based on the principle that hemodynamic changes often precede symptoms by days to weeks. However, device-based intrathoracic impedance monitoring alone has shown limited diagnostic performance (high false positive burden) and, in some settings, increased healthcare utilization without clear outcome benefit [98]. Accordingly, digital CRT care is shifting toward multiparameter approaches that combine several device diagnostics (arrhythmia burden, ventricular rate during atrial arrhythmias, ventricular arrhythmias, activity trends, heart rate profiles/variability, and impedance) to improve short-term risk stratification for HF events [98]. Multisensor algorithms (composite indices incorporating heart sounds, respiration, thoracic impedance, heart rate, and activity) have demonstrated the ability to provide early warning of impending HF events in prospective validation studies, although outcome benefit depends on how alerts are operationalized in routine care and requires robust integration strategies [98]. In practical terms, integrating CRT with digital health is best framed as a care-delivery enabler: remote monitoring can support early troubleshooting of reduced biventricular pacing percentage, prompt review of evolving congestion patterns, and telemedicine-enabled medication optimization. Future needs include standardized alert-to-action pathways, minimization of false positives, and clarity on which combinations of sensor signals and management protocols improve hard outcomes.

## 9. Conclusions

CRT is associated with favorable multiorgan changes spanning neurohormonal, inflammatory/metabolic, gut axis, and renal/hepatic domains. Nevertheless, these effects are most plausibly interpreted as secondary consequences of improved cardiac performance and decongestion, and they appear predominantly responder-dependent. Because current evidence is largely observational and heterogeneous, it remains uncertain whether any changes reflect direct effects of CRT. Prospective studies with standardized hemodynamic and biomarker phenotyping are needed to clarify mechanisms and validate organ-axis trajectories as monitoring or therapeutic targets.

## Figures and Tables

**Table 1:** Clinical benefits of CRT according to major clinical trials.

Study	Population	Comparator	Key Outcomes	Main Findings
MUSTIC	Advanced HF, wide QRS	OMT/crossover	QoL, exercise capacity	Early proof-of-concept showing improved symptoms and functional capacity with CRT
MIRACLE	NYHA III–IV, reduced LVEF, wide QRS	OMT	QoL, 6MWD, NYHA class	Significant improvements in symptoms, exercise tolerance, and QoL Established symptomatic benefit
COMPANION	Advanced HF	OMT vs. CRT-P vs. CRT-D	Death or hospitalization, mortality	Both CRT arms reduced death or hospitalization CRT-D reduced all-cause mortality
CARE-HF	NYHA III–IV, reduced LVEF, dyssynchrony	OMT	Mortality, morbidity, LV remodeling	Landmark trial showing reduced mortality and HF hospitalizations
MADIT-CRT	Predominantly NYHA I–II, reduced LVEF	ICD alone	HF events, mortality	Reduced HF events (≈30–40%), especially in LBBB and QRS ≥ 150 ms No mortality difference
RAFT	NYHA II–III, reduced LVEF	ICD alone	Death or HF hospitalization	Reduced mortality and HF hospitalizations Higher device-related complications
REVERSE	Mild or asymptomatic HF	OMT	LV remodeling, HF progression	Significant LV reverse remodeling and delayed HF progression No mortality benefit
Adapt Response	NYHA II–IV, contemporary therapy	Adaptive vs. conventional CRT	Mortality	Very low mortality rates; benefit likely amplified by modern guideline-directed medical therapy

CRT, cardiac resynchronization therapy; CRT-D, cardiac resynchronization therapy with defibrillator; CRT-P, cardiac resynchronization therapy with pacing only; HF, heart failure; ICD, implantable cardioverter-defibrillator; LBBB, left bundle branch block; LVEF, left ventricular ejection fraction; NYHA, New York Heart Association; OMT, optimal medical therapy; QoL, quality of life; QRS, QRS duration on electrocardiogram; 6MWD, 6 min walk distance.

## Data Availability

No new data were created or analyzed in this study.
